# Novel Human-like Influenza A Viruses Circulate in Swine in Mexico and Chile

**DOI:** 10.1371/currents.outbreaks.c8b3207c9bad98474eca3013fa933ca6

**Published:** 2015-08-13

**Authors:** Martha Nelson, Marie R. Culhane, Albert Rovira, Montserrat Torremorell, Pedro Guerrero, Julio Norambuena

**Affiliations:** Fogarty International Center, National Institutes of Health, Bethesda, Maryland, USA; College of Veterinary Medicine, University of Minnesota, St. Paul, Minnesota, USA; College of Veterinary Medicine, University of Minnesota, St. Paul, Minnesota, USA; College of Veterinary Medicine, University of Minnesota, St. Paul, Minnesota, USA; ASPROCER, Santiago, Chile; ASPROCER, Santiago, Chile

## Abstract

Introduction: Further understanding of the genetic diversity and evolution of influenza A viruses circulating in swine (IAV-S) is important for the development of effective vaccines and our knowledge of pandemic threats. Until recently, very little was known of IAV-S diversity in Latin America, owing to a lack of surveillance.

Methods: To address this gap, we sequenced and conducted a phylogenetic analysis of 69 hemagglutinin (HA) sequences from IAV-S isolates collected in swine in Mexico and Chile during 2010-2014, including the H1N1, H1N2, and H3N2 subtypes.

Results: Our analysis identified multiple IAV-S lineages that appear to have been circulating undetected in swine for decades, including four novel IAV-S lineages of human seasonal virus origin that have not been previously identified in any swine populations globally. We also found evidence of repeated introductions of pandemic H1N1 viruses from humans into swine in Mexico and Chile since 2009, and incursions of H1 and H3 viruses from North American swine into Mexico.

Discussion: Overall, our findings indicate that at least 12 genetically distinct HA lineages circulate in Latin American swine herds, only two of which have been found in North American swine herds. Human-to-swine transmission, spatial migration via swine movements, and genomic reassortment are the key evolutionary mechanisms that generate this viral diversity. Additional antigenic characterization and whole-genome sequencing is greatly needed to understand the diversity and independent evolution of IAV-S in Latin America.

## Introduction

Since the 2009 H1N1 pandemic, our understanding of the global diversity and evolution of influenza A viruses in swine (IAV-S) has increased. A more complete picture of the genetic diversity of IAV-S circulating globally has been provided by the expansion of IAV-S surveillance in many countries, including Argentina^1^, Australia^2^, Cameroon^3^, Canada^4^, China^5^, Peru^6^, Japan^7^, Mexico^8^ , Sri Lanka^9^, Thailand^10^, the United States^11^, Vietnam^12^, and multiple countries in Europe^13^^,^ 14 ^,^ 15. The expansion of surveillance has advanced our understanding of how IAV diversity evolves in swine hosts, including the importance of frequent transmission of IAVs from humans to swine^16^^,^ 17, reassortment^5^^,^ 11 ^,^ 13 ^,^ 15 ^,^ 18 ^,^ 19, and the role of the live swine trade in disseminating viral diversity between countries and continents^20^^,^ 21. However, it remains unknown where the pandemic H1N1 (H1N1pdm09) virus evolved in swine for approximately a decade prior to its emergence in humans in 2009^22^, underscoring gaps in our knowledge of IAV-S spatial distribution, diversity, and evolution on a global scale. The fact that the first major outbreak of the H1N1pdm09 virus occurred in humans in Mexico^23^ underscores the importance of improving our knowledge IAV-S diversity in Latin America, where IAV-S surveillance has been historically low.

Recently increased IAV-S surveillance in Argentina, Brazil, Colombia, and Mexico has identified multiple IAV-S lineages of human origin, including pandemic and seasonal IAVs as well as reassortants^1^^,^ 6 ^,^ 8 ^,^ 24 ^,^ 25 ^,^ 26 ^,^ 27. In Argentina, IAVs of human origin have been identified in swine, including the H1N1, H1N2, and H3N2 subtypes, with evidence of circulation for many years prior to detection^16^^,^ 24. Viruses of human origin have been identified in swine in Brazil, including the H1N2 and H3N2 subtypes, again with evidence of long-term transmission in swine prior to their recent detection^28^. Viruses of human H1N1pdm09 origin also have been identified in swine in Colombia, Costa Rica, Cuba, and Mexico^28^^,^ 29. To date, there is no evidence of IAV-S disseminating between countries in Latin America^28^. It is also notable that although multiple genetically diverse HA and NA surface antigens of human origin have been identified in pigs in Latin America, the internal gene segments identified to date in Latin American swine have all been H1N1pdm09 in origin, evidence of frequent reassortment between H1N1pdm09 and other IAV-S lineages since 2009^24^^,^ 28.

To further understand the genetic diversity of IAV-S in Latin America, we sequenced the HA segments from 18 IAVs collected from swine in Chile in 2012 and 51 IAVs collected from swine in Mexico during 2010-2014 and performed a phylogenetic analysis using a Bayesian approach. We identified novel IAVs of human origin in pigs in both countries, as well as evidence of incursions of North American classical swine viruses from the United States (or Canada) into Mexico.

## Materials and Methods

**Data collection and sequencing in Chile**. A cross-sectional sampling from 22 production companies representing over 95% of commercial swine production in Chile were collected from July to October of 2012. Samples were collected from pigs in at least 32 farms distributed across 5 regions (Metropolitan Region and Regions III, VI, VII and IX). Two to five 8- to 12-week-old pigs showing subclinical and clinical signs of influenza-like illness (i.e., fever, nasal secretions and/or cough) were selected in each farm, necropsied and lung samples and tracheal swabs submitted to the University of Minnesota Veterinary Diagnostic Laboratory (VDL) for IAV-S matrix gene testing using a real-time RT-PCR^30^, and virus isolation in Madin Darby Canine Kidney (MDCK) cells^31^ of any IAV-S PCR positive samples. HA and NA subtyping of IAV-S RT-PCR positive samples was completed. HA gene sequences were either obtained from virus isolates or directly from the originally submitted respiratory tract material. To isolate viruses, the swab or tissue supernatant (in 400-μl amounts) was inoculated on monolayers of MDCK cells grown in 25-cm2 flasksd with 5 ml of MEM+ media. All cultures were incubated at 37°C under a 5% CO2 atmosphere. All flasks were examined daily for 7 days under an inverted light microscope to observe virus-induced cytopathic effects (CPE). Of the 91 lung samples submitted from samples from Chile, 50 tested positive by RT-PCR, yielding 26 isolates of the H1N1, H1N2 and H3N2 subtype. RNA was extracted from the original material or virus isolates as previously described. Briefly, viral RNA was extracted from 50 μl of swab supernatant using a magnetic bead procedure (Ambion® MagMAX™ AM1835 and AM1836, Applied Biosystems, Foster City, CA). Segment specific PCR fragments were obtained with One-Step RT-PCR (Qiagen, CA) using influenza A specific primers for HA, previously described elsewhere^32^. A total of 18 HA sequences were obtained (Table 1).

**Data collection and sequencing in Mexico**. Respiratory tract samples were collected by veterinarians and farm staff from pigs with clinical signs of respiratory disease on farms in Mexico between 2010 and 2014. These samples were sent ad hoc to the VDL for swine respiratory disease diagnostic investigations. At the VDL, all samples were tested as described above for samples from Chile. Virus isolation and viral sequencing were performed on influenza-positive samples as requested by the submitting party (Table 1).

**Submission of sequence data to GenBank. **All nucleotide sequences generated for this study have been submitted to the publicly accessible Influenza Virus Resource database at GenBank, with accession numbers KR870242-KR870310 (http://www.ncbi.nlm.nih.gov/genomes/FLU/Database/). All alignments are available from the authors upon request.

**Table 1. Characteristics of IAV-S from Chile and Mexico sequenced for this study.** Virus name, source of sample, collection date, and lineage (see Results below) for 69 influenza viruses collected from swine in Chile and Mexico during 2010-2014 that were sequenced for this study.

Name	Source	Date	Lineage
A/sw/Chile/9271613/2012/H1N2	tracheal swab	8/23/2012	Chile H1 human I
A/sw/Chile/9313899/2012/H1N1	lung	8/8/12	Chile H1 human I
A/sw/Chile/9313897/2012/H1N1	lung	8/8/12	Chile H1 human I
A/sw/Chile/9313898/2012/H1N1	lung	8/8/12	Chile H1 human I
A/sw/Chile/9210585/2012/H1N1	lung	7/25/12	Chile H1 human II
A/sw/Chile/9271617/2012/H1N2	tracheal swab	8/23/12	Chile H1 human II
A/sw/Chile/9271633/2012/H1N1	tracheal swab	8/23/12	Chile H1 human II
A/sw/Chile/9271642/2012/H1N2	tracheal swab	8/23/12	Chile H1 human II
A/sw/Chile/9271644/2012/H1	lung	8/23/12	Chile H1 human II
A/sw/Chile/9313895/2012/H1	lung	8/8/12	Chile H1 human II
A/sw/Chile/9313893/2012/H3N2	lung	8/xx/12	Chile H3 human I
A/sw/Chile/9313892/2012/H3N2	lung	7/xx/12	Chile H3 human II
A/sw/Mexico/9557133/2013/H1N1	lung	1/14/13	Mexico classical I
A/sw/Mexico/9762101/2013/H1N1	lung	4/30/13	Mexico classical I
A/sw/Mexico/9783445/2013/H1N1	lung	5/9/13	Mexico classical I
A/sw/Mexico/9800323/2013/H1N1	lung	5/16/13	Mexico classical I
A/sw/Mexico/8935602/2012/H1N1	lung	3/18/12	Mexico classical singleton
A/sw/Mexico/9453694/2012/H3N2	lung	11/22/12	Mexico H3 human
A/sw/Mexico/9634750/2013/H3N2	lung	2/24/13	Mexico H3 human
A/sw/Mexico/9684432/2013/H3N2	lung	3/21/13	Mexico H3 human
A/sw/Mexico/9714185/2013/H3N2	lung	4/7/13	Mexico H3 human
A/sw/Mexico/9741876/2013/H3N2	lung	4/21/13	Mexico H3 human
A/sw/Mexico/9783514/2013/H3N2	lung	5/9/13	Mexico H3 human
A/sw/Mexico/9857788/2013/H3N2	nasal swab	6/13/13	Mexico H3 human
A/sw/Mexico/10499819/2014/H3N2	nasal swab	4/27/14	Mexico H3-IV
A/sw/Mexico/7773583/2010/H3N2	nasal swab	9/15/10	Mexico H3-IV
A/sw/Mexico/7932475/2010/H3N2	nasal swab	11/28/10	Mexico H3-IV
A/sw/Mexico/8531936/2011/H3N2	oral fluids	9/1/11	Mexico H3-IV
A/sw/Mexico/8630459/2011/H3N2	nasal swab	10/20/11	Mexico H3-IV
A/sw/Mexico/8630565/2011/H3N2	nasal swab	10/20/11	Mexico H3-IV
A/sw/Mexico/8630588/2011/H3N2	nasal swab	10/20/11	Mexico H3-IV
A/sw/Mexico/8630638/2011/H3N2	nasal swab	10/20/11	Mexico H3-IV
A/sw/Mexico/9562931/2013/H3N1	lung	1/16/13	Mexico H3-IV
A/sw/Mexico/9783350/2013/H3N2	lung	5/9/13	Mexico H3-IV
A/sw/Mexico/9834542/2013/H3N2	oral fluids	6/3/13	Mexico H3-IV
A/sw/Mexico/9834545/2013/H3N2	oral fluids	6/3/13	Mexico H3-IV
A/sw/Mexico/9834567/2013/H3N2	oral fluids	6/3/13	Mexico H3-IV
A/sw/Mexico/9890611/2013/H3N2	oral fluids	6/30/13	Mexico H3-IV
A/sw/Chile/9313891/2012/H1N1	lung	9/13/12	Pandemic – Chile 1
A/sw/Chile/9313894/2012/H1N1	tracheal swab	7/xx/12	Pandemic – Chile 2
A/sw/Chile/9313896/2012/H1	lung	7/xx/12	Pandemic – Chile 2
A/sw/Chile/9210582/2012/H1N1	lung	7/25/12	Pandemic – Chile 3
A/sw/Chile/9271625/2012/H1	lung	8/23/12	Pandemic – Chile 4
A/sw/Chile/9210581/2012/H1N2	lung	7/25/12	Pandemic – Chile 5
A/sw/Mexico/9199572/2012/H1N1	lung	7/22/12	Pandemic – Mexico 1
A/sw/Mexico/9213802/2012/H1N1	lung	7/29/12	Pandemic – Mexico 1
A/sw/Mexico/9213809/2012/H1N1	oral fluids	7/29/12	Pandemic – Mexico 1
A/sw/Mexico/9722783/2013/H1N1	lung	4/10/13	Pandemic – Mexico 1
A/sw/Mexico/9741916/2013/H1N1	lung	4/21/13	Pandemic – Mexico 1
A/sw/Mexico/9800380/2013/H1N1	lung	5/16/13	Pandemic – Mexico 1
A/sw/Mexico/9800385/2013/H1N1	lung	5/16/13	Pandemic – Mexico 1
A/sw/Mexico/9800390/2013/H1N1	lung	5/16/13	Pandemic – Mexico 1
A/sw/Mexico/9922474/2013/H1N1	nasal swab	7/16/13	Pandemic – Mexico 1
A/sw/Mexico/9713846/2013/H1N1	lung	4/7/13	Pandemic – Mexico 1
A/sw/Mexico/9425974/2012/H1N1	lung	11/7/12	Pandemic – Mexico 3
A/sw/Mexico/10032432/2013/H1N2	lung	9/9/13	Pandemic – Mexico 4
A/sw/Mexico/10290795/2014/H1N2	lung	1/15/14	Pandemic – Mexico 4
A/sw/Mexico/9711817/2013/H1N2	lung	4/4/13	Pandemic – Mexico 4
A/sw/Mexico/9800379/2013/H1N2	nasal swab	5/16/13	Pandemic – Mexico 4
A/sw/Mexico/9812228/2013/H1N2	oral fluids	5/22/13	Pandemic – Mexico 4
A/sw/Mexico/7942103/2010/H1N1	lung	12/1/10	Pandemic – Mexico 5
A/sw/Mexico/8630529/2011/H1N1	nasal swab	10/20/11	Pandemic – Mexico 5
A/sw/Mexico/8630550/2011/H1N1	nasal swab	10/20/11	Pandemic – Mexico 5
A/sw/Mexico/9583196/2013/H1N1	lung	1/28/13	Pandemic – Mexico 5
A/sw/Mexico/7774063/2010/H1N1	nasal swab	9/15/10	Pandemic – Mexico 6
A/sw/Mexico/10126158/2013/H1N1	lung	10/24/13	Pandemic – Mexico 7
A/sw/Mexico/10248405/2013/H1N1	nasal swab	12/25/13	Pandemic – Mexico 7
A/sw/Mexico/10433030/2014/H1N1	lung	3/25/14	Pandemic – Mexico 7
A/sw/Mexico/10466772/2014/H1N1	lung	4/9/14	Pandemic – Mexico 7

**Phylogenetic analysis**. Nucleotide alignments were generated for the H3 and H1 segments, separately, using MUSCLE v3.8.31^33^. An initial H1 tree was inferred using neighbor-joining (NJ) methods to categorize the H1 segments as either classical swine virus, human pandemic, or human seasonal virus origin. As global background data, related human and swine H1 and H3 sequences were downloaded from the Influenza Virus Resource^34^ that were studied previously^16^^,^ 20 (Table S1). The final alignments consisted of (a) 357 classical swine H1 sequences (5 IAV-S sequenced for this study from swine in Mexico and 321 classical swine H1 viruses collected globally that have been studied previously, and 31 pandemic viruses included as reference), (b) 491 human and human-origin swine H3 sequences (21 viruses sequenced for this study from swine in Mexico, 2 viruses sequenced for this study from swine in Chile, 256 human seasonal H3N2 viruses collected globally during 1968 – 2013, and 212 closely related swine H3 viruses collected globally that have been studied previously); (c) 204 human and human-origin swine H1 sequences (10 viruses sequenced for this study from swine in Chile, 108 human seasonal H1 viruses collected globally during 1978 – 2009, and 86 closely related IAV-S isolates collected globally that have been studied previously); and (d) 998 pandemic H1N1 viruses (6 viruses sequenced for this study from swine in Chile, 25 viruses sequenced for this study from swine in Mexico, 212 IAV-S isolates collected globally, and 760 human pandemic H1N1 viruses collected globally during 2009-2014).

For each of the four final alignments, the phylogenetic relationships were inferred using the maximum likelihood (ML) method available in the program RAxML v7.2.6^35^, incorporating a general time-reversible (GTR) model of nucleotide substitution with a gamma-distributed rate variation among sites. To assess the robustness of each node, a bootstrap resampling process was performed (500 replicates). We utilized the high-performance computational capabilities of the Biowulf Linux cluster at the National Institutes of Health, Bethesda, MD (http://biowulf.nih.gov). Time-scaled phylogenies were inferred using the MCMC methods available in the BEAST package v1.8.0^36^, using a relaxed uncorrelated lognormal (UCLN) molecular clock, a flexible Bayesian skyline plot (BSP) demographic model (10 piece-wise constant groups), and a general-time reversible (GTR) model of nucleotide substitution with a gamma-distributed rate variation among sites. The MCMC chain was run separately three times for each of the data sets for at least 100 million iterations, with sub-sampling every 10,000 iterations. The BEAGLE library^37^ was used to improve computational performance. All parameters reached convergence, as assessed visually using Tracer v.1.6, with statistical uncertainty reflected by values of the 95% highest posterior density (HPD). The initial 10% of the chain was removed as burn-in, runs were combined using LogCombiner v1.8.0, and maximum clade credibility (MCC) trees were summarized using TreeAnnotator v1.8.0.

**Estimating the timing of viral introductions in Chile and Mexico.** The timing of viral introductions into swine in Chile and Mexico from outside sources (humans, North American swine, etc.) was inferred from time-scaled Bayesian MCC trees, based on the times to the Most Recent Common Ancestor (tMRCAs) of two nodes on the phylogeny, including the 95% HPD: the node where a Mexican or Chilean swine clade coalesces (i.e., the tMRCA of that clade) and the node where the clade coalesces with the background human or US swine viruses. Extremely long branch lengths representing gaps in data complicate estimation of the timing of viral introductions into Chile and Mexico and result in estimates with wide ranges reflecting this uncertainty. We conservatively report these full ranges, but also acknowledge that the likelihood of unsampled viruses being of human origin, US IAV-S origin, or Latin American origin is related to the intensity of IAV surveillance in these three populations. Surveillance of human IAVs is orders of magnitude greater than surveillance in swine, and it is more likely that long branches represent unsampled IAV-S than unsampled human viruses. Following this reasoning, it is also less likely that IAVs circulated undetected for many decades in the historically more intensively sampled US swine herds than in Latin America, where surveillance has only begun in recent years. We therefore report estimates of the full range of uncertainty, acknowledging that the lower-bound estimate is highly conservative, but also qualitatively consider the likelihood of estimates within this range based on knowledge of variations in surveillance intensities in different hosts and regions. In our inference of the spatial origins of IAV-S lineages in Latin America, we also consider IAV-S in the United States and Canada to represent a meta-population, based on previous findings^20^, and account for the possibility that a virus of apparent US origins may in fact be from less-sampled Canadian swine herds.

**Estimating genetic distances**. Pairwise genetic distances (Kimura 2-parameter) were estimated using MEGA5^38^ for representative IAV-S isolates from Chile and Mexico. Representative reference strains were selected for the H1a cluster (A/sw/Saskatchewan/01974/2008/H1N1), H1b (A/sw/Texas/SG1380/2011/H1N1), H1g (A/sw/Illinois/02251/2008/H1N1), H1pdm (A/sw/Chile/9210582/2012/H1N1), H3-IV (A/sw/Ontario/33853/2005/H3N2), original triple reassortant viruses (A/sw/Minnesota/593/99/H3N2), H1d1 (A/sw/Oklahoma/053259/2008/H1N2), H1d2 (A/sw/Arkansas/02927/2009/H1N2), representative viruses from Brazil and Argentina (A/wild boar/Brazil/214-11-13D/2011/H1N2, A/sw/Brazil/365/11-7-2011/H3N2, A/sw/Argentina/CIP051-BsAs76/2009/H1N1), and the most closely related human viruses (A/Suita/1/1989/H1N1, A/Texas/36/1991/H1N1, and A/New/York/700/1995/H3N2).

## Results

**Genetic diversity of IAV-S in Mexico and Chile**. Among the 51 IAVs sequenced from swine in Mexico and 18 IAVs sequenced from swine in Chile, extensive genetic diversity was observed in the HA segment, including viruses related to the (i) classical North American H1 viruses and H3-IV lineages, (ii) H1N1pdm09 lineage, (iii) a genetically distinct H3 IAV-S lineage that has recently been identified in Brazilian swine^28^, and (iv) multiple novel IAV-S lineages in Mexico and Chile of human seasonal virus origin that have not been previously characterized in any other country to date (Tables 1 and 2). Several of these novel IAV-S lineages are highly genetic divergent from all other IAVs identified in swine and humans globally to date (Table S2), and are separated by extremely long branch lengths representing multiple decades of independent evolution and major gaps in sampling (Figs. 1-3). These gaps in sampling complicate the estimation of the timing of viral introduction into Chile and Mexico from other sources (e.g., humans, US swine herds.). Conservatively, we report the full range of uncertainty (Table S3). However, we also recognize that the likelihood of unsampled viruses being of human origin, US IAV-S origin, or Latin American origin is inversely related to the intensity of IAV surveillance, and varies substantially among these three populations.

**Classical swine H1 influenza viruses Mexico**. Five IAVs collected from swine in Mexico were genetically related to the H1 classical swine virus lineage that has circulated in North American swine since the 1918 ‘Spanish flu’ pandemic (Tables 1 and 2). No IAVs collected in swine in Chile were related to classical H1 viruses. A phylogenetic tree including the 5 Mexican H1 viruses, as well as background classical H1 viruses sampled globally from swine, indicates that classical H1 viruses have been introduced into Mexico from the United States at least twice independently. One of these introductions only involves a singleton virus (A/sw/Mexico/8935602/2012/H1N1, Table 1) that is closely related to the North American H1b virus A/sw/Texas/SG1380/2011/H1N1 (99.8% genetic similarity, Table S2; Fig. 1; ML tree with tip labels displayed available in Fig. S1). The close genetic relationship between A/sw/Mexico/8935602/2012/H1N1 and A/sw/Texas/SG1380/2011/H1N1 indicates that this virus was likely introduced into Mexican swine relatively recently (2010.5 – 2012.2, 95% HPD, Table S3)

**Table 2. Influenza A viruses identified in swine in Chile and Mexico.** Introductions into swine in Chile and Mexico of influenza A viruses from North American swine (classical H1 and H3-IV viruses) and humans (pandemic H1N1 viruses and seasonal H1N1/H3N2 viruses). § One of these introductions is a singleton (A/sw/Mexico/8935602/2012/H1N1) with little evidence of onward transmission. ¶ It remains unclear whether one of these introductions (Chile H3 human II, A/sw/Chile/9313892/2012/H3N2) represents a separate introduction of seasonal H3N2 viruses from humans, or represents dissemination via swine of a previously described introduction of human H3N2 viruses into swine in Brazil.

	Classical H1 (North American swine)	H3-IV (North American swine)	Human pandemic H1	Human seasonal H1	Human seasonal H3
Chile	0	0	5	2	2¶
Mexico	2§	1	7	0	1

A second introduction of IAV-S from the US to Mexico involves a clade of four Mexican viruses collected in 2013 (‘Mexico Classical I’, Fig. 1, Table 1) that are not closely related to any other IAV-S in our global sampling, with > 10% genetic divergence from representative IAV-S sequences from North America (Table S2). Consistent with high genetic divergence, these viruses are separated phylogenetically by a long branch length representing approximately 20 years of independent evolution (~1991.7 – 2012.1, estimated times to the Most Recent Common Ancestor (tMRCAs) for the two nodes defining this phylogenetic branch, Table S3, Fig. 1). The most likely explanation is that these viruses represent a novel classical-like IAV-S lineage that was introduced from US (or Canadian) swine to Mexico several decades ago, as it is more likely that the long branch represents unsampled viruses in Mexico, owing to the greater intensity of surveillance in swine in the US than in Mexico.

**Phylogenetic relationships between classical swine H1 segments:**
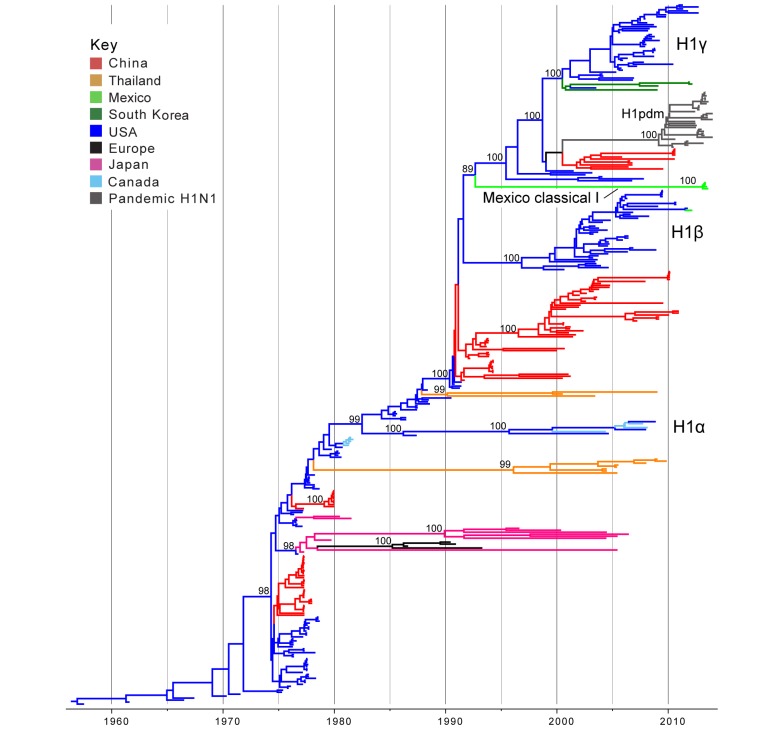
Time-scaled Bayesian MCC tree inferred for the HA (H1) sequences of 357 viruses, including 5 viruses sequenced for this study from swine in Mexico, 321 classical swine H1 viruses collected globally, and 31 pandemic viruses included as reference. Branches are shaded by country of origin: China = red; Thailand = orange; Mexico = light green; South Korea = dark green; USA/Canada = dark blue; Europe = black; and Japan = pink. As reference, pandemic H1N1 viruses are shaded dark grey. Posterior probabilities > 0.8 are included for key nodes. North American classical IAV-S lineages are labeled: H1α, H1β, and H1γ. The clade of Mexico classical I viruses is labeled (Table 1), and the clade of representative pandemic viruses is labeled ‘H1pdm’ (a detailed phylogeny of H1pdm evolution is available in Fig. S5). The tMRCAs representing the estimated timing of viral introduction into Mexico are provided in parentheses. A similar phylogeny inferred using ML methods with tip labels displayed is available in Fig. S1.

**Human-origin H3 influenza viruses in swine in Mexico and Chile**. Two IAVs collected from swine in Chile and 21 IAVs collected from swine in Mexico were related to human seasonal H3N2 viruses (Table 1). A time-scaled MCC phylogenetic tree including these 23 H3 sequences, as well as background H3 sequences from viruses sampled globally from humans and swine, indicates that the H3 viruses identified in Chile and Mexico cluster into four genetically distinct groups: two Mexican clades and two Chilean singletons (Fig. 2, ML tree with tip labels displayed available in Fig. S2). One clade containing 7 H3N2 viruses collected in swine in Mexico during 2012-2013 (‘Mexico H3 human’, Fig. 2, Table 1) represents a novel introduction of human seasonal H3N2 viruses into swine in Mexico. The introduction is estimated to have occurred up to ~20 years ago (~1994.8 – 2010.9, 95% HPD, Table S3). It is likely that the long branch length represents unsampled IAV-S rather than unsampled human viruses, given the greater intensity of IAV surveillance in humans. Therefore, it is likely that the human-to-swine transmission event occurred in the mid-1990s, similar to the timing of the major introduction of H3N2 triple reassortant viruses into US swine^39^, although represents an independent human-to-swine transmission event.

Fourteen of the H3N2 viruses collected in our study from swine in Mexico, sampled 2010-2014, are closely related to five other Mexican swine viruses that were characterized previously^20^. Consistent with previous findings, this cluster of 19 Mexican viruses is phylogenetically positioned within the H3-IV lineage, which has been the dominant H3 lineage in US swine herds in recent years (‘Mexico H3.IV’, Fig. 2, Tables 1 and 2). The posterior support for the node defining the entire cluster of all 19 Mexican H3-IV viruses is not high enough to be considered a true clade (75%). Sub-clades of Mexican viruses within the cluster are very highly supported (100%) and could be considered up to five independent introductions of H3-IV viruses from the United States into Mexico. However, the most parsimonious explanation is that these Mexican viruses represent a single IAV-S migration event from the US (or Canada) into Mexico via live swine movements, with onward dissemination and diversification into sub-lineages within pigs in Mexico. This introduction is estimated to have occurred during 2005.0 – 2005.5 (Table S3).

**Phylogenetic relationships between human and swine H3 segments:**
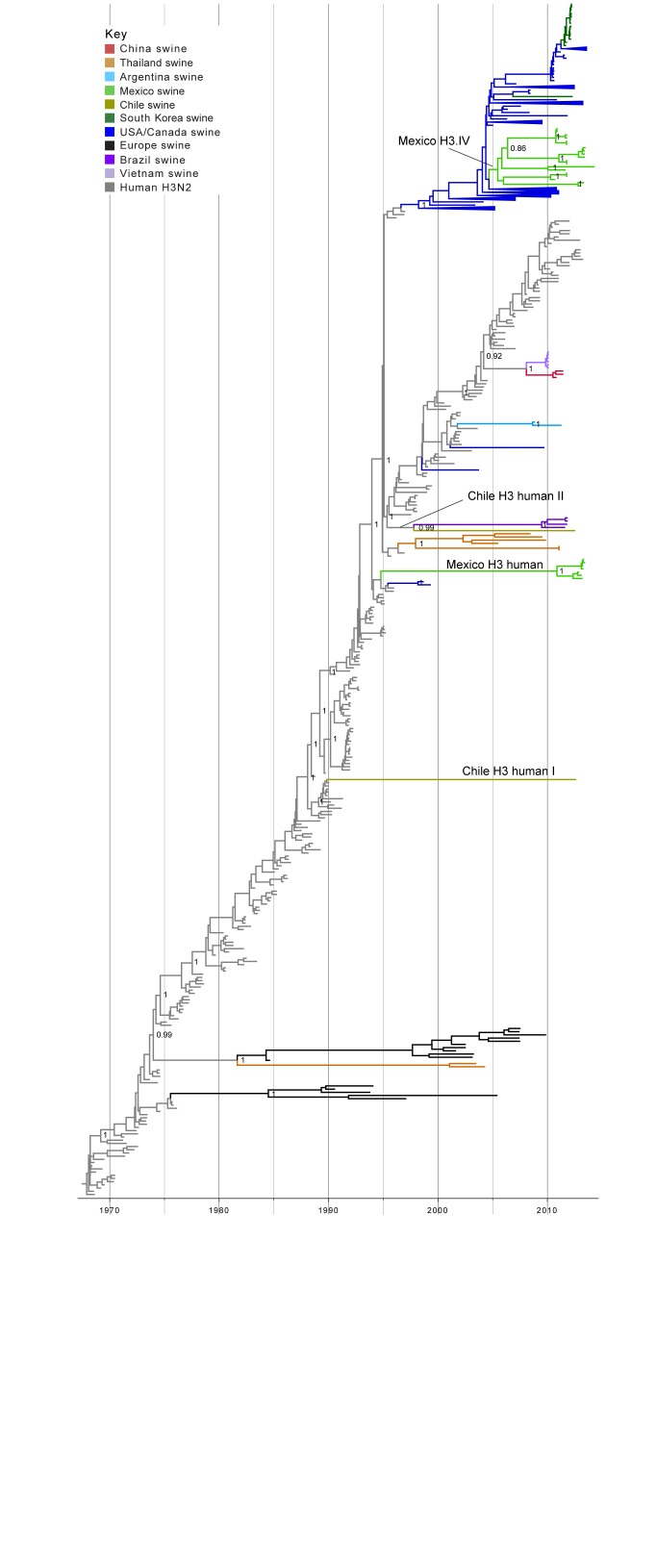
Time-scaled Bayesian MCC tree inferred for the HA (H3) sequences of 491 viruses, including 21 viruses sequenced for this study from swine in Mexico, 2 viruses sequenced for this study from swine in Chile, 256 human seasonal H3N2 viruses collected globally during 1968 – 2013, and 212 closely related swine H3 viruses collected globally. Branches of human seasonal H3 influenza virus origin are shaded grey, and branches associated with viruses from swine are shaded by country of origin, similar to Fig. 1, with the addition of: Argentina = light blue; Brazil = dark purple; and Vietnam = light purple. Posterior probabilities > 0.8 are included for key nodes. The posterior probability (0.75) also is provided for the cluster of 14 Mexico H3.IV viruses. The clade of 7 Mexico H3 human viruses, the Chile H3 human I singleton virus, and the Chile H3 human II virus that clusters with the Brazilian clade of IAV-S also are labeled (Table 1). The tMRCAs representing the estimated timing of viral introduction into Mexico and Chile are provided in parentheses. A similar phylogeny inferred using ML methods with tip labels displayed is available in Fig. S2.

In Chile, the singleton H3N2 virus A/sw/Chile/9313893/2012/H3N2 is separated by an extremely long branch length from all other IAV-S on the phylogeny (Chile H3 human I, Fig. 2), and has low genetic similarity with all other IAVs available from humans and swine (< 90%, Table S2). The phylogeny indicates that A/sw/Chile/9313893/2012/H3N2 represents another novel H3N2 IAV-S lineage that was introduced from humans into swine, in this case as many as ~25 years ago (1989.9 – 2012.7, Table S3). It is difficult to infer the evolutionary history of a singleton virus separated by such a long branch length. But again, differences in the intensity of IAV surveillance in humans and swine increases the likelihood that the long branch represents unsampled IAV-S, rather than human viruses. It is therefore most likely that this human-origin lineage circulated undetected in swine in Chile (or possibly other unsampled Latin American countries) for almost two and half decades.

A/sw/Chile/9313892/2012/H3N2 represents another independent introduction of H3N2 viruses from humans (‘Chile H3 human II’, Fig. 2). In this case, A/sw/Chile/9313892/2012/H3N2 is most closely related to a clade of four viruses that were identified in swine in Brazil (represented by A/sw/Brazil/365/11-7/2011/H3N2). On the MCC tree, these five viruses are together supported as a clade (99% posterior probability, Fig. 2), suggesting this group of Latin American viruses represents a single human-to-swine introduction, estimated to have occurred during ~1995.3 – 1997.8 (Table S3). A/sw/Chile/9313892/2012/H3N2 and the Brazilian IAV-S cluster still remain grouped together on a phylogeny that includes all available full-length H3 sequences from GenBank from human viruses collected during 1992-1997 (n = 454 H3 sequences, Fig. S3). However, bootstrap support for this grouping is low (43%, Fig. S2), and the Brazilian and Chilean viruses are not highly similar at a nucleotide level (89.5% genetic similarity, Table S2). It therefore is not possible without additional data to discern with certainty whether the Brazilian cluster and Chilean singleton represent two independent introductions of human viruses into swine, or a single human-to-swine introduction that many years ago disseminated from pigs in Brazil to Chile, or vice versa.

**Human seasonal H1 influenza viruses in swine in Chile**. Ten of the IAV-S isolates from Chilean pigs were related to human seasonal H1N1 viruses (Tables 1 and 2). No Mexican viruses were related to human seasonal H1N1 viruses. A phylogenetic tree including these 10 Chilean viruses, as well as background human seasonal H1 viruses and human-like swine H1 viruses sampled globally, indicates that the H1 viruses identified in Chile are not closely related to any other H1 viruses identified to date in swine (Fig. 3). Rather, the Chilean H1s are most closely related to human seasonal H1N1 viruses from the late 1980s and early 1990s, although separated by very long branch lengths. The Chilean H1s are positioned in two different sections of the tree, representing two independent introductions from humans (‘Chile H1 human I’ and ‘Chile H1 human II’, Fig. 3, Table 1, ML tree available in Fig. S4). The ‘Chile H1 human I’ clade is most closely related to human viruses from the late 1980s (e.g., A/Suita/1989/H1N1), and the ‘Chile H1 human II’ clade is most closely related to human viruses from the early 1990s (e.g., A/Texas/36/1991/H1N1) (Table S2, Figs. 3 and S4). Both clades are 100% supported and separated from the human viruses by long branch lengths representing over 15 years of evolution (1988.1 – 2012.6 and 1990.4 – 2005.9, respectively, Fig. 3, Table S3). The presence of the H1N2 subtype within both of these clades is evidence of reassortment events with the N2 segment since transmitting to swine.

**Phylogenetic relationships between human and swine H1 segments:**
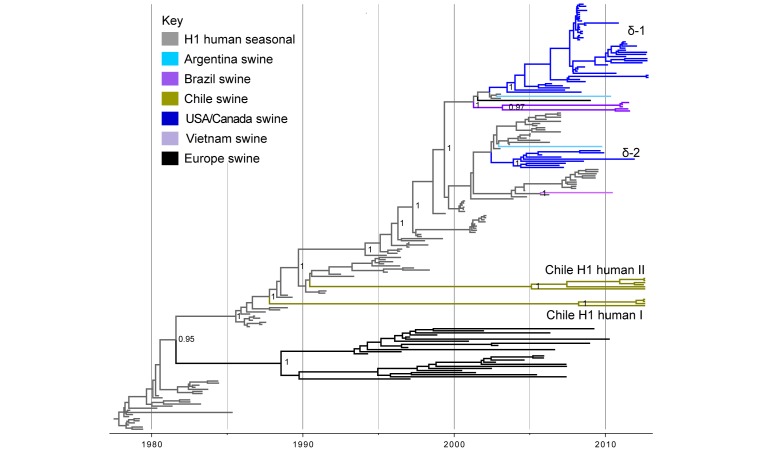
Time-scaled Bayesian MCC tree inferred for the HA (H1) sequences of 204 viruses, including 10 viruses sequenced for this study from swine in Chile, 108 human seasonal H1 viruses collected globally during 1978 – 2009, and 86 closely related IAV-S collected globally. Branches of human seasonal H1 influenza virus origin are shaded grey, and branches associated with viruses from swine are shaded by country of origin, similar to Figs. 1 and 2. Posterior probabilities > 0.8 are included for key nodes. The North American H1δ1 and H1δ2 lineages are labeled, as well as the clades of Chilean viruses (‘Chile H1 human I’ and ‘Chile H1 human II’, Table 1). The tMRCAs representing the estimated timing of viral introduction into Chile are provided in parentheses. A similar phylogeny inferred using ML methods with tip labels displayed is available in Fig. S4.

**Pandemic H1 influenza viruses in Mexico and Chile**. Six viruses from Chile and 25 viruses from Mexico were related to human H1N1pdm09 viruses (Tables 1 and 2). A phylogenetic tree including these 31 IAV-S, as well as background H1N1pdm09 viruses sampled globally from humans and swine, indicates that pandemic H1 viruses have been transmitted from humans into swine in Mexico at least 7 times, including the A/sw/04/Mexico/2009/H1N1 virus that has been studied previously^8^ (Fig. S5, Table 1). Based on phylogenetic position with respect to time-structured human diversity, 6 human-to-swine transmission events are estimated to have occurred during the initial waves of the H1N1 pandemic in humans that occurred in 2009-2010 (Mexico 1 – Mexico 6, Fig. S2), and one clade of Mexican IAV-S (Mexico 7, Fig. S2) clusters with human H1N1pdm09 viruses isolated in more recent years (e.g., A/California/19/2013/H1N1), indicative of a more recent human-to-swine transmission event. The population of IAV-S from Chile consists of four singletons and one clade on the phylogeny, indicative of five separate introductions from humans to swine (Fig. S5, Table 1). Although the pandemic IAV-S population from Chile was sampled in 2012, these IAVs also appear to have been introduced from humans during the initial pandemic wave of 2009-2010. Importantly, most of these viral introductions are separated by long branch lengths representing several years of circulation in swine, evidence of onward transmission of the H1N1pdm09 HA segment in swine in Mexico and Chile.

## Discussion

Through new surveillance efforts and phylogenetic analysis of IAV-S in Mexico and Chile, we have expanded our understanding of the extensive IAV-S diversity that circulates in swine in Latin America. Most notably, we have identified multiple novel clades of H3N2 and H1N1 viruses of human origin in Mexico and Chile that have not been identified in swine previously, highlighting the importance of the human-swine interface in the evolution of IAV-S diversity in Latin America. The presence of two different IAV-S lineages in Mexico that are related to North American IAV-S (classical H1N1 and H3-cluster IV) also demonstrates the importance of viral migration from US and/or Canadian swine herds into Mexico. The identification of a group of human-origin H3 IAV-S isolates from swine in both Brazil and Chile could be evidence of IAV-S migration between two countries in Latin America, but additional sequence data are needed to rule out the possibility of two independent human-to-swine transmissions in these respective countries. The fact that live swine are not frequently transported between Brazil and Chile supports the likelihood of two independent introductions from humans, but additional data are greatly needed. Consistent with patterns observed globally^16^, at least 12 introductions of human H1N1pdm09 viruses in to swine were observed in Chile and Mexico since 2009, further contributing to IAV-S diversity. Overall, we identified four key evolutionary processes that generate viral diversity in swine in Latin America: (a) human-to-swine transmission of pandemic influenza viruses, (b) human-to-swine transmission of seasonal influenza viruses, (c) introduction of viruses from US/Canadian swine into Mexico, and (d) reassortment.

The identification of multiple genetically diverse IAVs circulating in swine in Latin America complicates the development of effective vaccines in the region. Including our findings, at least 12 genetically distinct HA lineages have been identified in Latin American swine to date. Seven of these HA lineages have been identified in previous studies of Latin American IAV-S: (i) IAV-S of human seasonal H1N1 virus origin isolated in Argentina^24^, (ii) IAV-S of human seasonal H1N2 virus origin isolated in Argentina^24^, (iiI) IAV-S of human seasonal H1N2 virus origin isolated in Brazil^25^, (iv) IAV-S of human seasonal H3N2 virus origin isolated in Argentina^24^, (v) IAV-S of human seasonal H3N2 virus origin isolated in Brazil^28^, (vi) H3-cluster IV viruses of North American origin isolated in Mexico^20^, and (vii) H1N1pdm09 viruses in multiple countries in Latin America^1^^,^ 8 ^,^ 28 ^,^ 40. Our study describes at least five additional HA lineages in Latin America: (i) classical H1 IAV-S of North American origin isolated in Mexico (Mexico classical I), (ii-iii) two genetically distinct IAV-S lineages of human seasonal H1N1 virus origin isolated in Chile (Chile H1 human I and Chile H1 human II), (iv) IAV-S of human seasonal H3N2 virus origin isolated in Chile (Chile H3 human I), and (v) IAV-S of human seasonal H3N2 virus origin isolated in Mexico (Mexico H3 human). It is also possible that the other H3N2 singleton from Chile (Chile H3 human II) represents another independent IAV-S lineage of human origin.

It will be important to develop a standardized nomenclature to differentiate this multitude of novel HA lineages, many of which are not found in US swine herds or elsewhere globally. Several of these lineages are highly genetically divergent from IAV-S found in North America, and further antigenic characterization through hemagglutination inhibition (HI) assays is greatly needed. Although some NA segments have been sequenced in Argentina and Brazil, the genetic diversity of the NA in swine in Chile and Mexico is still not well understood and also requires further study. Due to the frequency of reassortment, whole-genome sequencing would be particularly useful for understanding the evolutionary dynamics of IAV-S in Chile and Mexico. The internal gene segments in other Latin American countries frequently have been found to be H1N1pdm09 in origin, owing to reassortment between H1N1pdm09 viruses with a diversity of HA and NA segments^16^.

Our findings are consistent with recently developed global models of IAV-S evolution in which humans are key sources of IAV-S diversity. In addition to the 20 lineages of human seasonal virus origin identified previously^16^, our study identified at least four additional novel IAV-S lineages of human seasonal virus origin: Mexico H3 human, Chile H1 human I, Chile H1 human II, and Chile H3 human I. Notably, all four of these introductions of human viruses into Latin American swine were estimated to have occurred over twenty years ago, suggesting that IAV-S has circulated undetected in both Mexico and Chile for decades. The southbound migration of IAV-S from swine in the US or Canada into Mexico, with no evidence of IAV-S migration in the northbound direction, also is consistent with the direction of the live swine trade and previous models of global IAV-S evolution^20^. Only two of the 12 IAV-S lineages in Latin America were related to known North American IAV-S lineages, indicating that IAV-S in Latin America for the most part are evolving independently from IAV-S in North America and require independent surveillance efforts.

No avian-like Eurasian IAV-S were identified in any Latin American countries to date, and it still remains unclear how or where the novel reassortant North American-Eurasian IAV-S that gave rise to the H1N1pdm09 virus evolved in swine prior to 2009. A reassortment event between North American and Eurasian IAV-S lineages is more likely to have occurred in swine in Asia^20^, where both North American and Eurasian viruses co-circulate and reassortants have been identified previously^41^. However, it remains unclear how a virus that most likely emerged in swine the Eastern hemisphere could have caused its first major outbreak in humans in Mexico. The diversity of IAV-S we identified in Mexico and Chile, even with relatively limited sampling, suggests that a great diversity IAV-S circulates in Latin American swine herds, including lineages that likely have not been detected to date, and expanded surveillance in the region is greatly needed.

## Competing interests

The authors have declared that no competing interests exist.

## Acknowledgements

We would like to thank all participating veterinarians and producers for providing the samples used in this study. In addition, we would like to thank Dr. Devi Patnayak, Lindsey Raymond, Wendy Wiese, and Lotus Smasal from the University of Minnesota Veterinary Diagnostic Lab for their assistance in sample preparation and sequencing.

## Supporting Information

Supplementary Materials
